# Genetic diversity of *Mycobacterium tuberculosis* complex strains isolated from livestock workers and cattle in Nigeria

**DOI:** 10.1371/journal.pone.0211637

**Published:** 2019-02-20

**Authors:** Hezekiah K. Adesokan, Elizabeth M. Streicher, Paul D. van Helden, Rob M. Warren, Simeon I. B. Cadmus

**Affiliations:** 1 Department of Veterinary Public Health and Preventive Medicine, University of Ibadan, Ibadan, Nigeria; 2 DST-NRF Centre of Excellence for Biomedical Tuberculosis Research/SAMRC Centre for Tuberculosis Research, Division of Molecular Biology and Human Genetics, Faculty of Medicine and Health Sciences, Stellenbosch University, Tygerberg, Cape Town, South Africa; 3 Centre for Control and Prevention of Zoonoses, Faculty of Veterinary Medicine, University of Ibadan, Ibadan, Nigeria; Bundeswehr Institute of Microbiology, GERMANY

## Abstract

Molecular typing techniques are useful in understanding tuberculosis epidemiology; yet, they have been under-utilised at the human-animal interface in Nigeria. Sixty-four *Mycobacterium tuberculosis* complex (MTBC) isolates including 42 *M*. *tuberculosis*, 13 *M*. *bovis* and nine *M*. *africanum* obtained from livestock workers (LW, n = 47) and their cattle (n = 17) in three geographical zones of Nigeria were genotyped to identify and evaluate the genetic diversity of the circulating MTBC using spoligotyping. Distribution into clades of *M*. *tuberculosis* revealed; 45.3% Uganda I- [SIT46- cattle: 1; LW: 28], 14.1% Latin American Mediterranean- [SIT61, cattle: 1; LW: 8], and 1.6% T- [SIT53—LW: 1]. The *M*. *bovis* strains were 6.3% SB0944 [cattle: 4] and 1.6% each of SB0300, SB1026, SB1027 and SB1439 [cattle: 4]. Seventeen MTBC isolates [cattle: 7; LW: 10] yielded 14 new spoligotype patterns including three *M*. *tuberculosis* strains (three isolates), five *M*. *bovis* strains (five isolates) and six *M*. *africanum* strains (nine isolates), two of which belonged to MAF1. Only few families namely, the not previously described Uganda I-, LAM and SB0944 are predominant among the LW and cattle, with other types in lower prevalences. The strain population structure indicates an intriguing diversity and possible zoonotic linkage with consequences for TB control in the country. The need to employ newer molecular techniques such as Mycobacterial Interspersed Repetitive Unit-Variable Number Tandem Repeats and whole genome sequence to decipher circulating MTBC strains in Nigeria is advocated.

## Introduction

Tuberculosis (TB) afflicts millions of people each year and the leading cause of death from a single infectious agent, ranking above HIV/AIDS in 2017 [1]. As one of the top ten high TB burden countries, Nigeria with an estimated population of over 191 million had a total case notification of 104 904 of which 96% and 4% were, respectively pulmonary and extra-pulmonary TB in 2018 [1]. Bovine TB due to *Mycobacterium bovis* remains a disease of significant economic importance in cattle in Nigeria and comes with serious public health concern. Zoonotic TB in humans is associated with consumption of unpasteurized milk which is still regarded as the most important route of exposure in developing countries [2]. However, transmission of contaminated air droplets through aerosols from infected animals with pulmonary TB is common particularly among the occupationally exposed groups such as pastoralists and abattoir workers who are constantly in close contact with animals [3]. Conversely, humans suffering from active TB, such as farm workers, constitute the main source of *M*. *tuberculosis* infection and disease in animals, including cattle [4] following contamination of environment with cough aerosol, infected urine, faeces or sputum [5].

Molecular typing of *Mycobacterium tuberculosis* complex (MTBC) is an important adjunct to TB control, with respect to monitoring disease transmission, detecting or confirming outbreaks and laboratory error/cross-contamination, as well as identifying the clonal spread of successful clones [6]. Among PCR-based typing techniques is spoligotyping. It is a widely used method for genotyping MTBC organisms [7] and a rapid, inexpensive option that can be used to search for a relationship between strains in an ecological setting [8]. This method forms the platform for the development of publicly available strain databases, such as SITVITWEB [9] and www.m.bovis.org.

The direct repeat regions used for spoligotyping represents the clustered regularly interspaced short palindromic repeats (CRISPRs) region of the MTBC; and this has helped greatly in molecular epidemiological investigations/studies across the world.

While there have been a few reports on molecular diversity of MTBC strains in Nigeria, most were based exclusively on human populations [10–11] and at restricted sampling locations [10, 12]. Only limited reports are available which have included humans and animals that were conducted in geographically distinct locations which have no reported interactions between each other [13–14]. This study was aimed at determining the genetic diversity of MTBC strains circulating at the livestock worker-cattle interface at different geographical locations in Nigeria.

## Methods

### Study design, population and setting

This cross-sectional study was conducted among livestock workers (LW), specifically herdsmen and abattoir workers, as well as slaughtered and sedentary cattle in Ogun, Ebonyi and Sokoto States, Nigeria. The geographical locations and population densities for the three states are Ogun (7^o^00’N 3^o^35’E; 220 persons/km^2^), Ebonyi (6^o^15’N 8^o^05’E; 286 persons/km^2^) and Sokoto (13^o^05’N 05^o^15’E; 170 persons/km^2^). The states represent three of the six geographical zones in the country. Each of the states was purposively selected from each of the geographical regions as they represent states with a relatively high livestock population, human-animal interactions and carcass processing activities. Again, there is limited information on the incidence of TB in these locations in both cattle and humans. The herdsmen and abattoir workers in these regions like other developing countries are known to engage in activities that could enhance TB transmission at the human-animal interface, including living a communal lifestyle associated with overcrowding, consumption of raw unpasteurized cow milk and co-habitation with cattle.

### Ethical consideration and sample collection

The ethical approvals for procedures involving humans and cattle were respectively obtained from the University of Ibadan/University College Hospital Ethics Committee with approval number: NHREC/05/01/2008a and the University of Ibadan Animal Care and Use Research Ethics Committee with approval number: UI-ACUREC/App/2015/072. The isolates included in this study were earlier identified by the authors at the Tuberculosis Laboratory of the Department of Veterinary Public Health and Preventive Medicine, University of Ibadan, Nigeria as *Mycobacterium tuberculosis* complex according to a previously described deletion typing technique [15] from LW and cattle in the three states. Briefly, the reagents used for the PCR reaction were Q-Buffer, 10xBuffer, 25mM Mgcl_2_, 2.5mMdNTPs, and the primers used include RD1A, RD1B, RD1C, RD4A, RD4B, RD4C, RD9A, RD9B, RD9C, RD12A, RD12B and RD12C. All these with HotStarTag, isolate DNA and distilled water were added together and mixed for the running of the PCR reaction. Each PCR reaction contained 1μl DNA template, 5 μl Q-buffer, 2.5 μl 10 Xbuffer, 2 μl 25 mM MgCl_2_, 4 μl 10 mM dNTPs, 0.5 μl of each primer (50 pmol/μl), 0.125 μl HotStarTag DNA polymerase (Qiagen, Hilden, Germany) and was made up to 25 μl with water. Amplification was initiated by incubation at 95°C for 15 minutes followed by 45 cycles at 94°C for 1 minute, 62°C for 1 minute and 72°C for 1 minute. After the last cycle, the samples were incubated at 72°C for 10 minutes. PCR amplification products were electrophoretically fractionated in 3.0% agarose in 1Xtbe pH 8.3 at 6V/cm for 4 hours and visualised by staining with ethidium bromide.

These isolates were obtained between 2014 and 2015 from the sputum samples of 156 abattoir workers (Ogun: 76, Ebonyi: 21, Sokoto: 59) and 102 lesions of slaughtered cattle (Ogun: 86, Ebonyi: 7, Sokoto: 9) out of 1, 572 inspected (Ogun: 574, Ebonyi: 396, Sokoto: 602) at each state central abattoir as well as from sputum of 193 herdsmen (Ogun: 27, Ebonyi: 128, Sokoto: 38) and 333 milk samples (Ogun: 114, Ebonyi: 144, Sokoto: 75) from lactating cows from nine, fifteen and seven herds from the three states, respectively for the purposes of this study following Becton Dickinson digestion and decontamination procedure (BBL^TM^ MycoPrep^TM^ Kit, BD, Sparks, MD, USA) [16]. Following detailed explanation of the objectives and benefits of the study to the potential participants, we obtained oral consent from the participants since the literacy level of most of them was low. This was done by documenting each participant’s affirmation to partake in the study in the research book containing details of the different locations and codes to identify each participant. The University of Ibadan Ethics Committees approved using oral consents among populations such as livestock workers. The selected livestock workers and cattle herds were based on the number of consenting livestock workers and accessible herds at the time of sampling. Sampling of slaughtered cattle was based on selection of 15% of the total slaughtered for inspection during the study period. The sampling across the three states was predicated upon possible subclinical infection among livestock workers considering their unguarded close interactions with their cattle. There was no prior information regarding any evidence of on-going clinical infection or outbreak either among the livestock workers or their cattle. However, the setting is known for poor health care-seeking behaviour and classified as neglected and hard-to-reach communities. The concentrate thus obtained from the digestion and decontamination of the samples was inoculated onto Löwenstein-Jensen slopes with pyruvate and/or glycerol and incubated at 37°C for 12 weeks. Isolates were harvested for deletion typing and spoligotyping by scraping the growth from a slope into 200 μl of 7H9 Middlebrook (broth) and heating at 80°C for 1 h. From 71 available isolates, a total of 64 with detailed information connecting LW and their respective cattle together in each region were selected based on the geographic regions from where these samples were collected.

### Spoligotyping

Spoligotyping was carried out at Stellenbosch University, South Africa. This was done according to an established procedure [17] involving the use of a commercially available spoligotyping kit (Isogen, Bioscience BV, Maarssen, The Netherlands). This PCR-based fingerprinting method enables detection of the presence or absence of 43 variable spacer sequences located between short direct repeat (DR) sequences in the *M*. *tuberculosis* genome. Positive controls were the DNA from reference *M*. *tuberculosis* H37Rv and *M*. *bovis* BCG clones; while autoclaved ultrapure water was used as a negative control.

#### Comparison of spoligotypes with an updated database and data analysis

The identification of spoligotype profiles as well as their family was obtained by comparison with profiles deposited in the SITVIT2 (for *M*. *tuberculosis* and *M*. *africanum*) and www.m.bovis.org (for *M*. *bovis* strains). The spoligotypes of the *M*. *tuberculosis* and *M*. *africanum* strains were entered in the SITVIT2 database (Pasteur Institute of Guadeloupe, France), which is an updated version of the previously released SpolDB4 database [18]. In this database, SIT designates spoligotypes shared by two or more patient isolates, as opposed to “orphan” which designates patterns reported for a single isolate. Major phylogenetic clades were assigned according to signatures provided in the database, which defined 62 genetic lineages/sub-lineages [18]. Similarly, the spoligotypes of the *M*. *bovis* strains were compared with profiles deposited in the www.m.bovis.org database [19].

## Results

**Fig 1:**
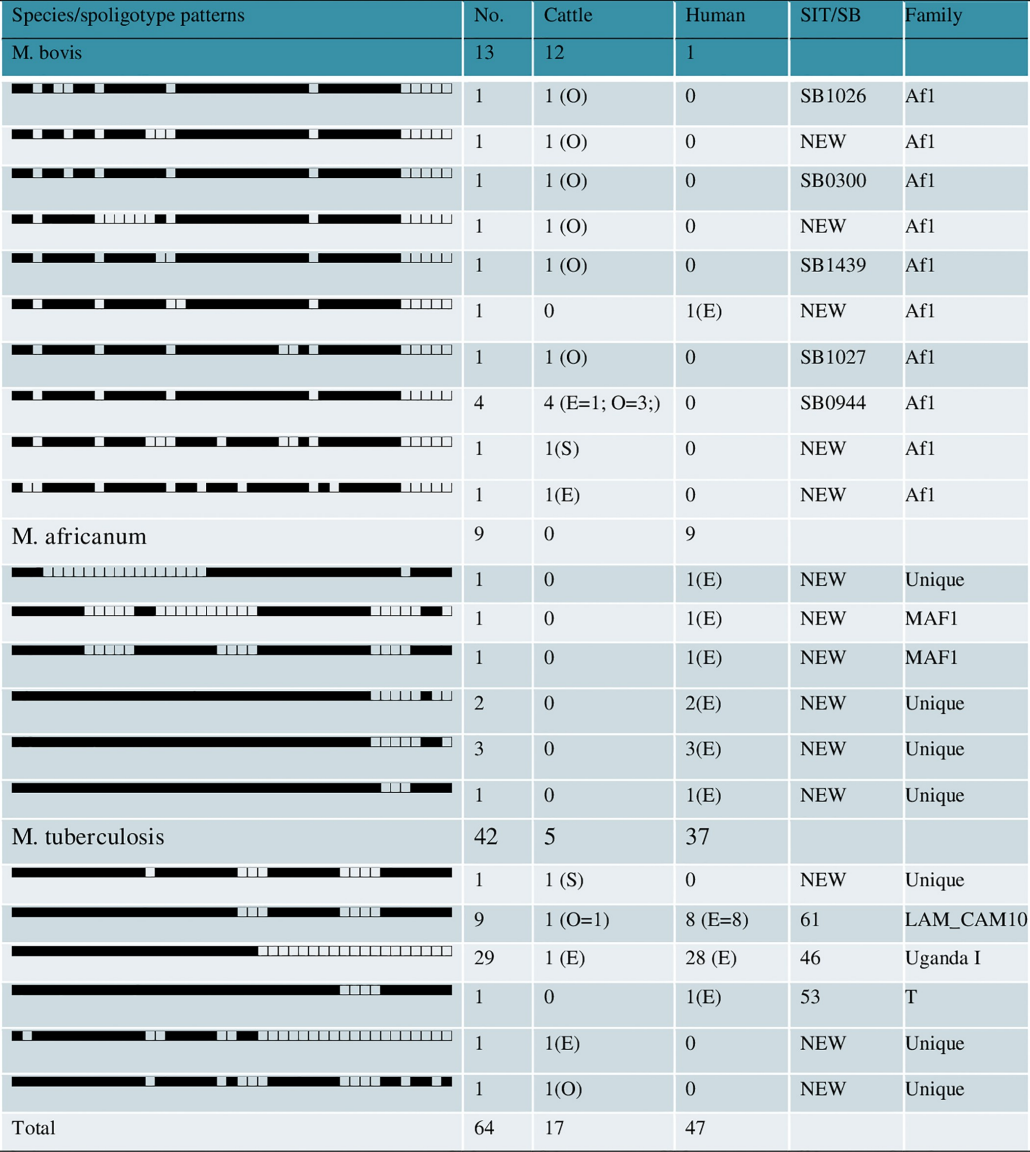
Spoligotype signatures of *Mycobacterium tuberculosis* complex isolates from cattle and livestock workers in Nigeria. Keys: O–Ogun; E–Ebonyi; S–Sokoto.

Of the 64 MTBC isolates genotyped (LW: sputum = 47, cattle = 17: milk = 2, lesion = 15), 42 were *M*. *tuberculosis*, 13 were *M*. *bovis* and 9 *M*. *africanum*. The results show that 47 (73.4%) of the isolates exhibited known spoligotype patterns (Fig 1). A total of 22 different spoligotype patterns were obtained giving an overall diversity (number of spoligotypes divided by the number of isolates) of 34.4. A total of 17 patterns occurred only once while there were five different clusters (n = 47) ranging from two to 29 isolates per cluster, suggesting a high clustering rate of 73.4%. Distribution into *M*. *tuberculosis* families revealed 45.3% Uganda I- [SIT46- cattle: milk = 1; LW: sputum = 28], 14.1% Latin American Mediterranean- [SIT61, cattle: lesion = 1; LW: sputum = 8], and 1.6% T- [SIT53—LW: sputum = 1]. The *M*. *bovis* strains were 6.3% SB0944 [cattle: lesion = 4], and 1.6% each of SB0300, SB1026, SB1027 and SB1439 [cattle: lesion = 4]. Fourteen new MTBC spoligotype patterns were identified [LW: sputum = 10; cattle = 7: milk = 1, lesion = 6] comprising three new *M*. *tuberculosis* strains (three isolates), six *M*. *africanum* strains (nine isolates) and five *M*. *bovis* strains (five isolates) (Fig 1).

### Analysis based on the region and type of samples collected

**Fig 2:**
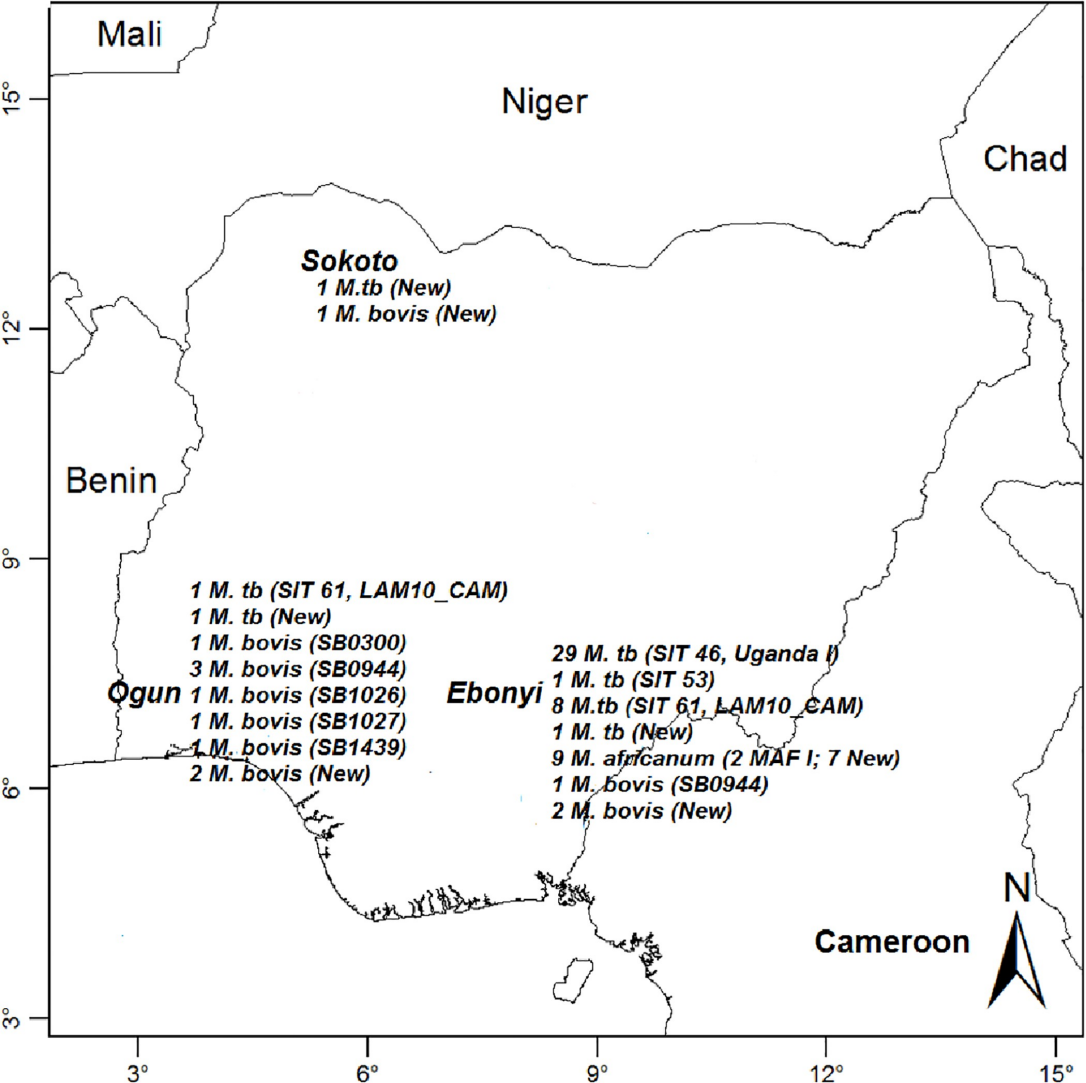
Spoligotype patterns of *Mycobacterium tuberculosis* complex strains from cattle and livestock workers from the three states in Nigeria.

From Ebonyi State, 39 *M*. *tuberculosis* (cattle = 2: milk = 1, lesion = 1; LW: sputum = 37) were identified by spoligotyping. Of these, 29 isolates (cattle: milk = 1; LW: sputum = 28) shared the same spoligotype pattern, SIT 46 of the Uganda I family, suggestive of zoonotic linkage. Other patterns obtained included SIT 61 of LAM_CAM10 family (LW: sputum = 8), SIT 53 of the T family (LW: sputum = 1) and a new strain (cattle: lesion = 1). In addition, three *M*. *bovis* belonging to SB0944 (cattle: lesion = 1) and two new strains (cattle: milk = 1, LW: sputum = 1) as well as nine *M*. *africanum* (LW: sputum = 9), two of which belonged to MAF1 were identified (Table 1, Fig 2). In Ogun State, two *M*. *tuberculosis* belonging to SIT 61 of LAM_CAM 10 family (cattle: lesion = 1) and a new strain (cattle: lesion = 1) as well as nine *M*. *bovis* (cattle: lesion = 9) of SB0944 (3 isolates), one isolate each of SB0300, SB1026, SB1027 and SB1439 and two new strains were identified. The two isolates identified by spoligotyping in Sokoto State were new strains of *M*. *tuberculosis* and *M*. *bovis* from slaughtered cattle (Table 1, Fig 2).

**Table 1:** Distribution of spoligotype patterns of *Mycobacterium tuberculosis* complex from cattle and LW from the three states in Nigeria.

State	Sample category	*M*. *tuberculosis* SITs				*M*. *africanum*	M. bovis SBs					
		46	53	61	New		0300	0944	1026	1027	1439	New
Ogun	Cattle	0	0	1	1	0	1	3	1	1	1	2
	*LW	0	0	0	0	0	0	0	0	0	0	0
Ebonyi	Cattle	1	0	0	1	0	0	1	0	0	0	1
	LW	28	1	8	0	9	0	0	0	0	0	1
Sokoto	Cattle	0	0	0	1	0	0	0	0	0	0	1
	LW	0	0	0	0	0	0	0	0	0	0	0
Total		29	1	9	3	9	1	4	1	1	1	5

* LW: Livestock workers

## Discussion

This study to our knowledge is the first molecular epidemiological investigations of MTBC among LW and cattle across three different geographical regions in Nigeria. We performed spoligotyping on 64 MTBC isolates to decipher the population structure of the MTBC strains of TB cases in LW and cattle.

We report for the first time SIT46 of the Uganda I family in Nigeria, a rare lineage. Assam *et al*. [20] reported that 8% of the MTBC strains causing disease in TB infected humans in Cameroon were due to Uganda I, with Cameroon being the closest border country to Nigeria among countries with reports of previous isolation of this strain. Koro *et al*. [21] also documented four strains of *M*. *tuberculosis* belonging to Uganda I from slaughter cattle in Cameroon. While Asante-Poku *et al*. [22] reported Uganda I strain in Ghana, the researchers showed that up to 65% of the TB cases in the country were due to Cameroon sub-lineage confirming an earlier report [23]. Hence, the possibility of this strain emanating from Cameroon, being the only country which shares some common border with Nigeria among the countries with available reports on the strain cannot be ruled out. Cameroon has a strong historical connection with Nigeria through family, business and animal trading system. Hence, the possibility of transmitting this strain from Cameroon to cattle or their handlers during trans-border activities between the countries is high.

The abundance of Uganda I, despite being isolated for the first time in the country, could be an indicator that the lineage is emerging in Ebonyi State and holds an important consideration in the epidemiology of TB in the state. In addition, considering its predominance and the fact that it was restricted to Ebonyi State, an on-going outbreak of TB due to this strain among the livestock workers in the state is likely. This becomes more of public health concern judging from the observation that the same strain was isolated from one of the cattle of these LWs in the state. This finding, thus suggests a possible reverse zoonotic linkage between livestock workers and their cattle, considering the risk factors such as cohabitation with cattle which could enhance transmission of the strain from infected person to cattle. This observation is a matter of public health concern since livestock workers especially the herdsmen and their cattle do move from one place to the other in search of water and pasture. This practice therefore provides a milieu for the spread of this strain of *M*. *tuberculosis* among human and animal populations.

Previous reports showed the LAM10-CAM family as the most predominant circulating clade in Nigeria [10–11, 13, 24]. Although this family was the next most abundant strain found in this study, our observation of Uganda I strain as predominant in the present study does not necessarily indicate the Uganda I strain as predominating in Nigeria considering the fact that it was being reported for the first time and more importantly was restricted to only Ebonyi State unlike LAM10-CAM which cuts across the states. However, this proportion might not be due to outbreak as the only strain isolated from cattle was from Ogun State when compared to eight other strains from livestock workers in Ebonyi of a different geographical location and mutually exclusive environment. The LAM10-CAM was first described in Cameroon, where it represented 34% of the *M*. *tuberculosis* isolates in 2003 [25] and has recently emerged as a dominant strain in the West province of Cameroon. This strain has also been reported in Senegal and Ivory Coast [25] and said to be dominant in other neighbouring West African countries, including the Republic of Chad, Benin, Burkina Faso and Ghana [22, 26–28] which share maritime borders and transhumance activities with Nigeria. Human and animal movement across the boundaries of these countries is substantial and constant, thereby corroborating the observation of a stable association of specific clones with geographically localized population [29].

Only one isolate exhibited the ill-defined T1 family in this study. This relatively low proportion has been previously observed in Nigeria. For instance, a comparatively lower 11% prevalence of T family than 66% LAM_CAM was reported in Anambra State [24], while 4% of genotyped *M*. *tuberculosis* in Jos belonged to the T family compared with the abundant 76% LAM_CAM family [10]. This lineage has also been reported among HIV positive TB patients in Nigeria [12]. Our finding therefore further reiterates the relatively low T family prevalence in the country.

The study also observed nine *M*. *africanum* isolates categorized into two (two spoligotype patterns) lacking spacers 8–12 and 37–39; thus, belonging to *M*. *africanum* West African 1 (MAF1) [30], five (two spoligotype patterns) exhibiting a partial signature of MAF1 (absence of spacers 37–39 but presence of spacers 8–12) [31] and two unknowns. The 19.1% prevalence of *M*. *africanum* among the strains confirmed by spoligotyping shows that this MTBC member remains an important cause of TB in humans. This is comparable to 13% reported in Ibadan, south-western Nigeria [13], but lower than 60% reported among infected humans from Guinea-Bissau [32]. This notwithstanding, the proportion of *M*. *africanum* in Western Africa, and particularly in Guinea–Bissau is diminishing with a more recent report showing 47.1% [33]. However, this apparent diminished reported incidence in certain regions of Africa as the cause of human TB is plausibly attributed to misidentification of *M*. *africanum* due to substantial phenotypic heterogeneity with some strains resembling *M*. *bovis* and others resembling *M*. *tuberculosis*. Although past studies [13, 34] have reported *M*. *africanum* in cattle, this species was not detected from cattle in this study. Previous reports have also indicated that this strain has been rarely isolated from this host [35–36] and it remains unclear whether it is also transmitted among cattle [34]. A more extensive study investigating *M*. *africanum* epidemiology in cattle in Nigeria is thus recommended.

In this study, we observed high clustering rates of spoligotypes (73.4%). This observation is in line with other reports in Nigeria [10, 24]. Considering independent mutational events associated with same spacer loss displayed by the spacers used in spoligotyping, the technique is not sufficient to establish epidemiological links and transmission analysis due to its limited discriminatory power [37]. Nevertheless, the high clustering rate detected in this study suggests rapid transmission of *M*. *tuberculosis* clones, particularly of the rare Uganda I lineage.

Another important observation in this study is the 26.6% of genotypes with no SITs or SBs in the international spoligotype databases [18], possibly reflecting micro evolutionary events in the DR region of an existing strain. This finding is similar to the report of Mbugi *et al*. [38] who observed new strains of *M*. *tuberculosis* circulating in the Serengeti ecosystem in Tanzania. Genomic diversity in MTBC remains a significant factor in TB pathogenesis that may affect virulence, transmissibility, host response and emergence of drug resistance [39]. As previously reported, some modern strains of MTBC such as the Beijing, Euro-American, Haarlem are believed to demonstrate more virulent phenotypes compared to ancient ones such as East African, Indian, *M*. *africanum* [39]. Based on this assertion therefore, the relatively high proportion of new strains in this study has implications on the epidemiology and control of TB in Nigeria. This becomes very important considering livestock workers and cattle movement especially from the northern region of the country down south in search of water and pasture during dry season. Also, livestock workers down south do go up north sourcing for animals for their herds. Such movement patterns provide a common point of inter-transmission of MTBC strains between the regions.

It is also remarkable that high number of workers were TB-infected in Ebonyi State compared to Ogun State (37 vs 0 *M*. *tuberculosis* isolates, respectively); whereas, two *M*. *tuberculosis* each were found in cattle from the two states. However, it must be noted that the two *M*. *tuberculosis* obtained in cattle from Ebonyi State were from milk from cattle herds as against the two *M*. *tuberculosis* from cattle lesions in Ogun State. Culturally, herdsmen live together in settlements and as communities. This communal lifestyle as well as cohabitation with their animals, result into longer contact time with one another and with their cattle, thus, enabling human-to-cattle, cattle-to-human and human-to-human TB transmission. On the other hand, abattoir workers spend lesser contact hours with one another as well as with slaughtered animals since they come from different places to meet at the abattoir. In addition, higher number of herdsmen were available for screening in Ebonyi State compared to relatively lower numbers of abattoir workers screened in Ogun State. Hence, possibilities of missed cases among abattoir workers in Ogun State could not be ruled out. This probably also highlights the low rate of transmission of *M*. *tuberculosis* in a cattle farm compared to the *M*. *bovis* infection. Then, the *M*. *tuberculosis* infection in this study is probably related to the direct/indirect contact of a TB-infected human with animals.

The isolation of different strains of *M*. *bovis* and *M*. *tuberculosis* from cattle in this study portends intra-transmission of these strains within cattle populations and inter-transmission between cattle and infected humans through both direct and reverse zoonotic transmission in the study area given the unguarded interactions which characterize the settings. The SB0944 constituted the most abundant spoligotype pattern of the *M*. *bovis* which is in agreement with the previous reports which indicated this spoligotype pattern as dominant in cattle in Nigeria [13] and Cameroon, a neighbouring country [40]. The isolation of *M*. *tuberculosis* in cattle is worth-noting; human-to-cattle transmission of *M*. *tuberculosis* has earlier been reported [13, 41], though it is generally believed that disease in cattle due to *M*. *tuberculosis* is less severe than that caused by *M*. *bovis*. This notwithstanding, the infection in cattle might result in reduced milk and meat yield with associated economic loss. Besides, the practice of pooling milk together in most developing countries could also lead to widespread infection among consumers since pasteurization of milk is not often enforced in these areas.

The above findings notwithstanding, the study had some limitations. One, sampling of the livestock workers and cattle herds was based on willingness and accessibility of livestock and their animals. This might have limited the numbers of people and cattle available for the study, thus preventing making robust epidemiological inferences. Two, only one state each was selected from three of the six geographical regions; sampling more states would have provided deeper insights into the circulating strains of MTBC in the country. Three, only spoligotyping was used to differentiate the isolated MTBC strains. The use of spoligotyping is known to possess limited discriminatory power compared to Mycobacterial Interspersed Repetitive Unit-Variable Number Tandem Repeats (MIRU-VNTR) and whole-genome sequencing. Despite these limitations, the present study has provided important insights into the circulating MTBC among livestock workers and cattle in Nigeria.

## Conclusions

This study provides information on the prevailing MTBC strains circulating among LW and cattle across three different states of Nigeria using molecular tool. We report a high prevalence of Uganda I strain, a rare lineage hitherto unreported in the country with a possible reverse zoonotic transmission of this strain between LW and cattle. Only few successful families including Uganda I, LAM_CAM10 of *M*. *tuberculosis* and SB0944 of *M*. *bovis* were predominant. Other families obtained were T family of *M*. *tuberculosis* and SB0300, SB1026, SB1027 and SB1439 of *M*. *bovis*. The finding of the big cluster of 29 isolates could indicate recent and probably rapid transmission and is therefore alarming. Hence, there is need to employ newer typing techniques such as MIRU-VNTR and whole-genome sequence to investigate the detected clusters for better epidemiological insights. Efforts are urgently needed to institute and enforce strict regulations against TB transmission in the country through a coordinated approach to regulate animal/LW movements particularly across borders, enforcement of pasteurization of milk, detailed routine meat inspection as well as public health enlightenment campaigns among LW. There is a need for a national policy geared towards mandating every LW and their cattle to undergo routine TB screening in order to prevent transmission of MTBC at the human-animal interface. This becomes urgently paramount given the currently reported poor practices [42]and the recently launched Road Map for Zoonotic Tuberculosis [43] which resonates the fact that controlling zoonotic TB is key to achieving the 2030 WHO’s End TB Strategy targeting TB elimination globally. Synergy between local and international agencies towards promoting capacity building and upgrading TB referral centres in the country to the level of using molecular tools in diagnosing TB rather than relying solely on smear microscopy is highly advocated.
